# Rapid molecular testing is associated with decreased broad-spectrum antibiotic use among patients with streptococcal bloodstream infections

**DOI:** 10.1017/ash.2024.430

**Published:** 2024-10-10

**Authors:** Tyler Tate, J. Alex Viehman, Ryan K. Shields

**Affiliations:** 1 Department of Medicine, Division of Infectious Diseases, University of Pittsburgh, Pittsburgh, PA, USA; 2 Antibiotic Management Program, University of Pittsburgh Medical Center, Pittsburgh, PA, USA; 3 Center for Innovative Antimicrobial Therapy, University of Pittsburgh, Pittsburgh, PA, USA

## Abstract

We investigated the impact of rapid diagnostic testing with real-time stewardship intervention on patients with *Streptococcal* bacteremia. Compared to pre-intervention, patients in the post-intervention group received more rapid antibiotic de-escalation (42 vs 88 h), and were more commonly de-escalated to narrow-spectrum (86% vs 52%) and oral antibiotics (30% vs 14%).

## Introduction

Rapid molecular testing allows antimicrobial stewardship programs (ASP) to intervene early in the setting of sepsis. Such approaches have led to lower rates of death and hospital re-admission among patients with bacteremia.^1^ Combining rapid diagnostic tests (RDT) with stewardship intervention further results in decreased time to antibiotic de-escalation and more efficient use of healthcare resources when compared to use of RDT without stewardship.^2–4^

The impact of RDT with stewardship intervention on contaminated blood cultures has not been fully defined.^5^ This is due, in part, to the diagnostic challenge of differentiating true bacteremia from potentially contaminated blood cultures in hospitalized patients. This paradigm applies to *Streptococcal* species identified in blood cultures, which represent a range of clinical syndromes from severe, life-threatening endovascular infections to contamination from normal skin flora.^6^ Thus, rapid initiation of antibiotic therapy can be life-saving or unnecessary. Given current rates of contamination, positive blood cultures are generally over-treated. This paradigm in most often described in the setting of coagulase-negative *Staphylococcal* bacteremia where up to 59% of patients receive unnecessary parenteral vancomycin following identification of a positive blood culture, which is associated with costs ranging from $2,397 to $11,152 per patient.^7^

Clinical recommendations have been proposed for antibiotic de-escalation based on RDT results;^8^ however, the use of RDT is streptococcal bacteremia is not well-established. Moreover, there are few data demonstrating the impact of ASP intervention for blood cultures with *Streptococcus* spp. and no data demonstrating the utility of RDT at the time of first positive blood culture when the diagnosis may not yet be clear.^5,9^ The objective of this study was to evaluate the clinical impact of RDT with real-time ASP intervention on blood cultures growing *Streptococcus* species.

## Methods

This study compared consecutive patients with *Streptococcus* species identified in blood cultures retrospectively over two 6-month periods. During the pre-intervention period (July–December 2019) our ASP monitored positive blood cultures using real time alerts Monday through Friday from 06:00 to 17:00. The team provided non-standardized recommendations following organism identification and susceptibility testing results, which were generally available 48–72 hours after Gram-stain results. During the post-intervention period all blood cultures were tested by GenMark Dx (member of the Roche group) ePlex^®^ Blood Culture Identification Gram-Positive panel (BCID-GP; Carlsbad, CA). Tests were run from 06:00 to 22:00, and results were reported directly to the ASP.^4^ Blood cultures that turned positive during the overnight period were tested the following morning. The panel identifies *S. agalactiae, S. anginosus* group, *S. pneumoniae,* and *S. pyogenes* along with all other streptococci (to the genus level), within 90 minutes from sample preparation. Upon receipt of results the ASP provided recommendations for antibiotic discontinuation, de-escalation, and additional diagnostic testing. We hypothesized that the duration of broad-spectrum antibiotic therapy would be shorter in the post-intervention period due to direct communication of results and earlier stewardship team involvement.

The primary endpoint of the study was time to antibiotic de-escalation, which was calculated as the time of the first dose of broad-spectrum antibiotic administration until the time of the first dose of de-escalated antibiotic therapy. Broad-spectrum treatment was defined as receipt of vancomycin, daptomycin, linezolid, piperacillin-tazobactam, or cefepime. Targeted therapy was defined as an antibiotic change made in response to the identified streptococcal organism. Secondary outcomes included hospital length of stay, development of acute kidney injury (defined by KDIGO criteria^10^), rate of re-infection with *Streptococcus* species and 90-day mortality. Blood cultures were classified as contaminants if the primary team discontinued antibiotic therapy and no signs/symptoms of bacteremia were evident. Statistical comparisons between pre- and post-intervention groups were made using Fisher’s exact test and Mann-Whitney U test for categorial and continuous variables, respectively. All data for this study were collected retrospectively with a waiver of informed consent granted by the University of Pittsburgh Institutional Review Board (STUDY 22070065).

## Results

**Figure 1.**
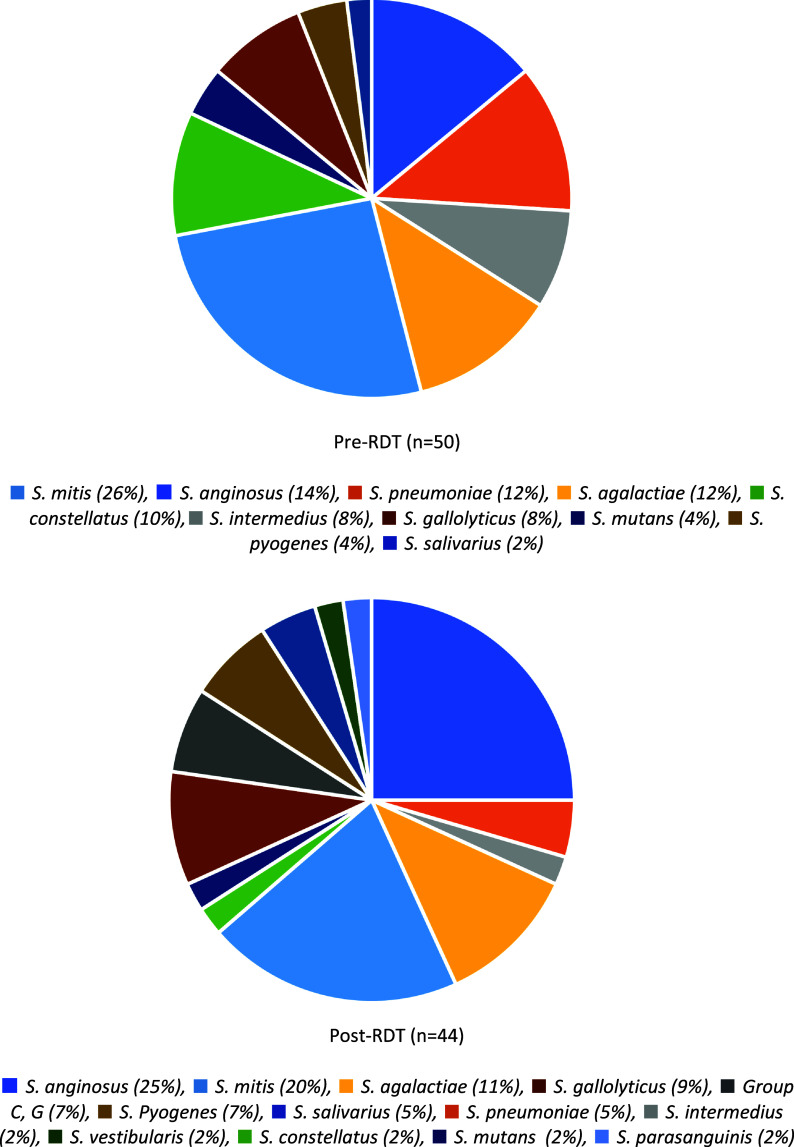
Blood culture results, pre- and post- rapid diagnostic testing.

Ninety-four patients were included in the analysis: 50 in the pre-RDT and 44 in the post-RDT periods. Across cohorts, patient demographics, clinical characteristics, and severity of illness were comparable (Table 1). The median age was 58 years, 65% were men, and 14% were immunocompromised. The mean qSOFA score was 2. Sources and complications of streptococcal bacteremia such as pneumonia or abscess formation were similar between groups; however, there was a trend toward a higher proportion of endocarditis in the post-RDT group (Table 1). The distribution of *Streptococcal* spp in the pre- and post-RDT periods is illustrated in the Figure 1. The most commonly administered empiric antibiotic both pre- and post-RDT was vancomycin (82%).

**Table 1.** Demographics, clinical characteristics and outcomes of patients with streptococcal bacteremia

	Pre-RDT (*n* = 50)	Post-RDT (*n* = 44)	*P* value
**Median Age (IQR)**	58 (44–69)	55 (42–69)	0.72
**Male**	31 (62%)	30 (68%)	0.67
**Solid organ transplant**	5 (10%)	3 (7%)	0.72
Immunocompromised status*	6 (12%)	7 (16%)	0.77
**Injection drug use**	8 (16%)	11 (25%)	0.31
**Presence of cardiac device**	7 (14%)	3 (7%)	0.33
**Noncardiac vascular device**	5 (10%)	5 (11%)	1.00
**Receiving parental nutrition**	1 (2%)	1 (2%)	1.00
**Diabetes**	18 (36%)	13 (30%)	0.52
**Renal replacement therapy**	4 (8%)	1 (2%)	0.37
**Solid tumor**	2 (4%)	5 (11%)	0.25
**Cirrhosis**	7 (14%)	8 (18%)	0.78
**Mean qSOFA score**	1.9	2	0.57
**ICU**	26 (52%)	17 (39%)	0.22
**Single positive blood culture bottle**	7 (14%)	1 (2%)	0.06
**Treated isolate as a contaminant**	6 (12%)	1 (2%)	0.12
**Presence of endocarditis**	2 (4%)	7 (14%)	0.08
**Abscess**	7 (14%)	6 (14%)	1.00
**Pneumonia**	9 (18%)	8 (18%)	1.00
**Social Factor**	11 (22%)	8 (18%)	0.80
**Concurrent infections**	18 (36%)	14 (32%)	0.83
Median time to de-escalation (Hours)**	88 (IQR 65 – 128)	42 (IQR 26 – 84)	0.002
**Targeted therapy**	26 (52%)	38 (86%)	0.0004
**Penicillin**	5 (19%)	8 (21%)	1
**Ampicillin**	2 (8%)	7 (18%)	0.3
**Cefazolin**	3 (12%)	5 (13%)	1
**Ceftriaxone**	14 (54%)	14 (37%)	0.2
**Other**	2 (8%)	4 (11%)	1
**AKI**	15 (30%)	11 (25%)	0.65
**Median LoS (days)**	9.0	9.5	0.27
**Alive at 30 days**	37 (74%)	39 (89%)	0.06
**Alive at 90 days**	35 (70%)	38 (86%)	0.04
**Readmission at 90 days**	22 (44%)	19 (43%)	1
**Transition to PO**	7 (14%)	13 (30%)	0.08

RDT, rapid diagnostic testing; qSOFA, quick sepsis related organ failure assessment; ICU, intensive care unit; RDT, rapid diagnostic testing; IQR, interquartile range; AKI, acute kidney injury; LoS, length of Stay; PO, by mouth; Social Factor, comfort measures or patient directed discharge; Concurrent Infections, coexisting infections in addition to streptococcal bacteremia

* Immunocompromised status was defined as chronic steroid use ≥20 mg/day, history of splenectomy, autoimmune disorder requiring treatment with immunosuppressive therapy, absolute neutrophil count <500cells/L, chemotherapy within 30 days, or acquired immunodeficiency syndrome (AIDS).

** One patient in each the pre- and post-intervention group is included in the analysis who had antibiotics de-escalated prior to the team determining the positive blood culture was likely a contaminant.

Prior to RDT, the median time to antibiotic de-escalation was 88 hours. Following implementation of RDT with ASP intervention, median time to de-escalation was 42 hours (*P* = 0.002). De-escalation to targeted therapy improved from 52% to 86% in post-RDT (*P <* 0.01). Transition to oral antibiotics increased from 14% to 30% (*P =* 0.08). There were no differences in hospital re-admission within 90 days or acute kidney injury while on antibiotic therapy. A trend toward decreased 30- (*P =* 0.06) and 90-day (*P =* 0.04) mortality was identified in the post-RDT period compared to the pre-RDT period (Table 1).

## Discussion

This study demonstrates that blood culture RDT combined with real-time ASP intervention improves time to antibiotic de-escalation in *streptococcal* bacteremia. Studies have demonstrated a similar effect in *Staphylococcus aureus* bacteremia,^9^ but less has been documented in the setting of *streptococcal* bacteremia, which represents a broad range of clinical syndromes. Our data show a significant reduction in time to de-escalation after the implementation of RDT, which was also associated with earlier initiation of targeted therapy and transitions to oral antibiotics. Several reasons for these improved rates can be proposed. In the pre-RDT group, there was not a uniform ASP intervention, which changed once RDT were implemented and providers were called directly with results and recommendations. As previously demonstrated, providers are more likely to make a change based on RDT when accompanied by ASP intervention.^2^ Additionally, there was a trend towards a higher proportion of patients pre-RDT requiring ICU stay. This may have impacted our observed decreased mortality rate in the post-RDT period. While this study was not powered to detect a mortality difference, a higher severity of illness may have influenced decisions to remain on broad spectrum therapy. Given that there was no difference in other secondary outcomes (AKI, length of stay or readmission), it is unlikely that this mortality difference would be replicated in larger studies.

The reduction in duration of broad-spectrum therapy was observed despite some differences between groups. While contamination was numerically more frequent pre-RDT, there were similar rates of patients requiring treatment for concurrent infections which may delay antibiotic de-escalation. Social factors that may alter treatment decisions were similar between groups and rapid antibiotic de-escalation was not associated with deleterious effects. Given that prolonged use of broad spectrum antibiotics increases the risk of antibiotic resistance, *Clostridioides difficile* infection, and central line complications,^11,12^ reducing time to targeted therapy by nearly 48 hours is likely associated with improved patient safety and lower costs.

More broadly, these data support the utility of RDT of blood cultures guided by ASP intervention. In our experience, clinicians can appropriately narrow from broad-spectrum Gram-positive therapy based on the identified S*treptococcus* species and knowledge of local resistance rates. For example, beta-hemolytic *Streptococcus* targets, S. *pyogenes* and *S. agalactiae*, can be treated with penicillin in appropriate patients. Given that other Streptococci may be less susceptible to penicillin, our approach is to de-escalate from a glycopeptide (or oxazolidinone) to ceftriaxone while awaiting susceptibility testing. Empiric de-escalation based on RDT results may not be appropriate in all patients. Patients with septic shock requiring ICU stay often remain on broad spectrum therapy to ensure no additional infections require broader coverage. Those with suspected concurrent infectious sources or those with infectious syndromes not fully explained by positive *Streptococcus* blood culture may require broad empiric therapy.

Given the retrospective nature of this study, contamination was determined by the treating clinician without standardized guidelines, which reflects real-world practice. Moreover, our study was not powered to detect differences in clinical outcomes between patients managed with or without RDT plus stewardship intervention. Future adequately powered studies that employ clear criteria of when to withhold therapy for probable contamination would be helpful to pursue additional decreases in antimicrobial usage. Other commercially available blood culture RDT have similar *Streptococcus* targets suggesting that our findings can be replicated at other hospitals using alternate systems. A key component, however, is ensuring that adequate stewardship resources are available to intervene in real-time based on RDT results to maximize the benefits of rapid testing in clinical practice.
